# The Use of Gutta-Percha as a Root-Canal Filling

**Published:** 1893-09

**Authors:** S. Freeman

**Affiliations:** New York


### THE USE OF GUTTA-PERCHA AS A ROOT-CANAL FILLING.

BY S. FREEMAN, D.D.S., NEW YORK.

In the reading of the March edition I notice Dr. Eddy's paper on the above subject, as well as the discussion thereon. In this paper I will not explain the different methods employed, nor the treatment prior to their filling, but will state my mode of filling in a very concise manner. After thoroughly treating and drying the root-canal, take a Dunn's syringe with a platina point, and inject a drop or two of a saturated solution of hydronaphthol and chloroform in the root-canal. Then take a gutta-percha cone, place it in the canal as near the apex as possible, where it dissolves; in other words make the chloro-percha in the root, then follow this with one cone after another until the canal is entirely filled. If any "chloro-percha" passes through the apex of the root, it no doubt becomes encysted. I have never had any trouble arising therefrom, and I am positive that I have passed some through the apex, because the patients have noticed it by describing to me a slight stinging sensation, which quickly subsides, and no future trouble arises. I have employed this method for a number of years, first using iodoform and chloroform instead of hydronaphthol and chloroform.

I find this method simple, clean, antiseptic, and effective.

